# Alcohol as a Panacea

**Published:** 1896-04

**Authors:** 


### Alcohol as a Panacea.

The following is from a recent lecture by a prominent physician who is not known as a reformer nor prominent in the temperance cause :

"Of what use is alcohol in medicine? We have a better heart tonic in digitalis ; as a lung tonic, a vastly better in strychnin; nitrate of amyl acts quicker; atropin warms up better. To tide over a dangerous time we would prefer iron, quinine and strychnin, concentrated food, and attention to hygiene. Is it capable of prolonging life in any way, or of assisting nature in throwing off the microbes of disease? The most extravagant claims have been made for it by the laity. It is the one panacea for all the ills to which human flesh is heir. But when we come to look at its effects in a fair and impartial manner, do not its claims as a therapeutic agent rest on a very unstable foundation? It prevents oxidation of the tissues. Physiologists tell us that when taken into the system the effect of alcohol on the red blood corpuscles—which are the oxygen carriers—is to lessen their power of giving off oxygen, and in this way the oxidation of tissue is interfered with. It does not require any great stretch of the imagination to see that while this condition of inactivity exists all the organs of the body must suffer, for any interference with the supply of oxygen will necessarily interfere with the evolution of force. In 'Flint's Physiology' it is said: 'Alcohol is capable of being absorbed and taken into the blood, but it passes out again unchanged. It cannot be regarded as an ailment, and hence cannot take the place of articles that are assimilated.' The fact that those who indulge in alcoholic liquors are unable to endure the same amount of fatigue is conclusive evidence that alcohol is in no sense a food. Men have been enabled to endure the extremes of heat and cold much better without alcoholic liquors of any kind than with them. The effect of alcohol on the human organism, in either health or disease, is unfavorable, as its use tends to increase the risk of infection in contagious diseases, and the progress is rendered more grave because of diminished resistance on the part of the organism in either health or disease. It is perfectly safe to predict that the time is not far distant when it will be as rare for a physician to prescribe alcohol as it is now for him to prescribe blood-letting, and when a healthy man will no more think of taking alcohol, with a view of preserving health, than he would strychnin for the same end."

